# Review of “*Digenetic Trematodes of Indian Marine Fishes*” by Rokkam Madhavi and Rodney Bray

**DOI:** 10.1186/s13071-019-3577-6

**Published:** 2019-06-24

**Authors:** Thomas H. Cribb

**Affiliations:** School of Biological Sciences, The University of Queensland, Brisbane, Australia

## Abstract

Madhavi R, Bray R:* Digenetic Trematodes of Indian Marine Fishes*. Springer; 2018, 693 pages. ISBN 978-94-024-1533-9.

## Review

There is an ever-increasing literature and commentary about global biodiversity—how much there might be, how fast we are characterising it relative to losing it through extinction, and revealing considerable angst about issues such as the recognition of species and cryptic species. These issues are as real for parasites as perhaps any other ecological group of organisms. Because parasites are small, hidden, and even ‘nasty’, their taxonomic characterisation started later than for many other groups (certainly generally later than for their hosts) and, undoubtedly, there remains a huge amount to be done for almost all groups. The species accumulation curve for marine digenean trematodes (for which the present book covers a geographical and host-related subset) is progressing upwards rapidly, although not quite as fast as in the 1970s and 1980s. No-one knows if this reflects a diminution of effort or a thinning of the undescribed fauna. The resource that generates this species accumulation curve, the World Register of Marine Species (WoRMS), represents an ever-improving resource that assists parasite taxonomists with taxonomic information (and more) on parasites and their hosts. Resources such as WoRMS combined with insights from molecular approaches make right now arguably one of the best times to be involved in parasite taxonomy: plenty left to do and tremendous resources with which to attack the issues.

Of course, with a build-up about the ‘best of times’, it is necessary to note that there is bad news as well. Some of the bad news is the impending loss (perhaps without replacement) of long-standing hard-won expertise represented by the likes of Rokkam Madhavi and Rodney Bray, the authors of this book. WoRMS also generates histograms of authors describing the most species during the last decade. Of the 30 most prolific, we have lost two and another 12 are far closer to the ends of their careers (many retired, including Madhavi and Bray) than to the start. Of the younger brigade, few are in the kinds of permanent positions that will allow them to make the kind of sustained contributions to improved understanding of marine digeneans that make a real difference. This brings us to Madhavi and Bray. Rokkam Madhavi has been publishing since 1968 (she started very young) and Bray since 1973. In this period, they have published hundreds of papers, principally on the digeneans of marine fishes, and in the case of Madhavi, Indian marine fishes. They thus have unrivalled credentials to summarise the challenging digenean fauna of those fishes.

The Introduction to this book tells us that the marine fish fauna of India stands at about 2564 species spread along coastlines covering about 7520 km. From these fishes and these localities there are about 600 digenean species known. This tome summarises this fauna with introductions to 32 families and keys to the genera, keys to the species, drawings of every species, diagnoses and comprehensive references to the literature. All the components are important, but the literature on Indian parasites can be especially difficult to obtain, so the comprehensiveness of the survey is of special value. It is often little appreciated, outside the field, just how much taxonomic hypotheses (i.e. the proposal of species and genus + species combinations) are subject to debate and change. In a system such as the Digenea, where the taxonomic structure is evolving rapidly, it is a struggle to keep pace with all the changes that have been made and to detect those that should be made. This volume is especially strong in having identified synonymies and taxonomic problems in general.

This book is not a rollicking read. It provides a clear introduction to digeneans and the Indian fauna and there are then nearly 700 pages of taxonomy, tables and references. This book is a critical resource for anyone concerned with the biodiversity of trematodes. If nothing else, I hope that it will encourage and enable more work on the Indian fauna; there is much to be done. However, the nature of the biogeography of the Indo-Pacific region means that the taxa summarised here have relevance far beyond the shores of India. I have not ‘read’ the book myself, but I have dipped into it many times since acquiring it. It lives on the shelf immediately above my head next to the texts of most immediate value to my work. It may not be in that category for many readers, but I strongly encourage its purchase for your libraries. It represents a summary and distillation of knowledge of a kind that is increasingly hard to come by.

## Abbreviation

WoRMS: World Register of Marine Species

